# New strategies against drug resistance to herpes simplex virus

**DOI:** 10.1038/ijos.2016.3

**Published:** 2016-03-25

**Authors:** Yu-Chen Jiang, Hui Feng, Yu-Chun Lin, Xiu-Rong Guo

**Affiliations:** 1 State Key Laboratory of Oral Disease, West China School of Stomatology, Sichuan University, Chengdu, China; 2 XiangYa Stomatological Hospital, Central South University, Changsha, China; 3 Center for Nanotechnology, Munster, Germany; 4 Institute for Nanobiomedical Technology and Membrane Biology, West China Medical School, Sichuan University, Chengdu, China

**Keywords:** new strategies, drug resistance, herpes simplex virus, Janus-type nucleoside analogues, lethal mutagenesis

## Abstract

Herpes simplex virus (HSV), a member of the Herpesviridae family, is a significant human pathogen that results in mucocutaneous lesions in the oral cavity or genital infections. Acyclovir (ACV) and related nucleoside analogues can successfully treat HSV infections, but the emergence of drug resistance to ACV has created a barrier for the treatment of HSV infections, especially in immunocompromised patients. There is an urgent need to explore new and effective tactics to circumvent drug resistance to HSV. This review summarises the current strategies in the development of new targets (the DNA helicase/primase (H/P) complex), new types of molecules (nature products) and new antiviral mechanisms (lethal mutagenesis of Janus-type nucleosides) to fight the drug resistance of HSV.

## Herpes simplex virus and drug resistance

### Herpes virus detection and risk

Viral diseases are the primary cause of death among human infectious diseases worldwide.^1^ Herpesviridae is a large family of DNA-containing viruses that result in human infections to varying extents. This family comprises eight members, which can be grouped into three subfamilies (α, β, and γ) based on biological and genomic similarities.^2^ Human α-herpes viruses include herpes simplex viruses (HSV-1, HSV-2) and varicella zoster virus (VZV).^3, 4, 5, 6, 7, 8^ HSV infections are among the most common human diseases, and 60%–95% of the population is infected by at least one of these viruses. HSV-1 is frequently associated with oral and perioral infections, and HSV-2 generally causes genital infections. HSV leads to diseases that range from mild conditions to severe infections, such as cold sores, keratitis, corneal blindness, and encephalitis. HSV infections increase the risk for developing human immunodeficiency virus (HIV) infection and contribute to the HIV epidemic. HSV can become latent and subsequently reactivate under certain circumstances, such as emotional stress, fever, and immunosuppression. VZV is the causative agent of chickenpox and shingles. The human β-herpes viruses include cytomegalovirus, HHV-6, and HHV-7.^9, 10, 11, 12, 13^ The γ-herpes viruses include Epstein–Barr virus (EBV) and Kaposi's sarcoma associated with herpesvirus.^14, 15, 16, 17^ Acyclovir (ACV) and related drugs for the treatment of HSV infectious diseases are overwhelmingly successful,^18, 19^ but these anti-herpes drugs have also selected for drug-resistant strains after long-term use.^20, 21, 22, 23^ Therefore, there is an emergent need to explore new strategies against drug-resistant HSV.

### Current drug and antiviral mechanisms

There are three classes of drugs approved for treatment of HSV infections, and all of which target viral DNA replication: acyclic guanosine analogues,^24, 25, 26, 27, 28, 29, 30, 31, 32^ acyclic nucleotide analogues,^33, 34, 35^ and pyrophosphate analogues^36^^–37^ (Table 1). Typical drugs from these three categories include valacyclovir (VCV), cidofovir, and foscarnet. ACV (9-(2-hydroxyethoxymethyl) guanine) has become a gold standard for prophylaxis and treatment of HSV infections since its introduction in the 1980s. Researchers have developed a series of anti-HSV nucleoside drugs, such as VCV, famciclovir, and ganciclovir, which are also first-line drug treatments for HSV infections.

**Figure 1:**
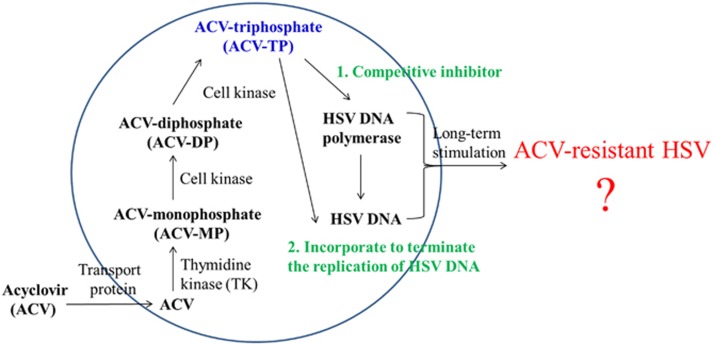
**The anti-HSV mechanism of ACV.** ACV, acyclovir; HSV, herpes simplex virus.

Nucleoside analogues have a similar anti-HSV mechanism. Nucleoside analogues, such as ACV, are selectively phosphorylated to a monophosphate derivative in infected cells by the virus-encoded thymidine kinase (TK). The affinity of ACV for HSV-TK is ~200 times greater than for human TK, and ACV displays remarkable safety against HSV. Cell kinases convert the monophosphate derivative of ACV to diphosphate- and triphosphate (TP)-active derivatives. The ACV-TP form is a competitive inhibitor of the viral DNA polymerase. ACV-TP also incorporates into the replicating DNA because of the absence of 3′ prime hydroxyl, which terminates the replication of viral DNA (refs. 2, 38–39) (Figure 1).

### ACV-resistant HSV

ACV and its derivatives are available for clinical application, and these agents are widely used for the treatment of HSV infections. However, long-term treatment with ACV and its derivatives may lead to drug resistance.^40, 41, 42, 43, 44, 45, 46^ A large difference has been observed between immunocompetent and immunocompromised patients. HSV infection in the former patients generally requires short-term anti-HSV therapy, and drug resistance does not easily occur. In contrast, the latter patients generally require long-term anti-HSV therapy, and they are likely to develop drug resistance. A low prevalence (range from 0.1% to 0.6%) of HSV resistance to ACV has been reported in normal immunocompetent patients. However, HSV resistance to ACV is more often isolated in immunocompromised patients and ranges from 3.5% to 10%.^22^ Some clinical surveys have reported a rate of ACV-resistant HSV isolates of up to 36%.^47^ For example, the prevalence of resistance to ACV among allogeneic bone marrow transplant patients has been reported to reach 30%.^48^ Resistant isolates result in severe, debilitating mucosal disease, and visceral dissemination. Therefore, the resistance of HSV to ACV is an important clinical problem for immunocompromised patients. The following resistance mechanisms of HSV to ACV have been reported:^2^ (a) decreased production of viral TK, (b) complete deficiency in viral TK activity, and (c) viral TK protein and DNA polymerase with altered substrate specify. The viral mutations conferring resistance to ACV are located in activating/phosphorylating genes (TK, *UL23* kinase) and the viral DNA pol enzyme (*UL30*), consistently with the above mechanisms of action.^42, 48, 49, 50, 51, 52^ The viral mutations in the TK gene generally result in incomplete or deficient enzymes because of the addition or deletion of nucleotides in long homopolymeric runs of Gs and Cs. Approximately 95% of ACV-resistant HSV clinical isolates have a TK-deficient phenotype. The target of anti-HSV drugs is primarily the DNA pol gene of HSV. A single mutation in DNA pol enzyme may confer resistance to many anti-HSV agents.^53, 54, 55^ For example, most ACV-resistant HSV isolates are also resistant to penciclovir because of a mutation in viral DNA polymerase.

## Strategies against drug resistance

Drug-resistant HSV mutants may result in more severe and chronic infections in immunocompromised patients, given the increasing numbers of transplant and cancer patients. Therefore, the emergence of drug-resistant HSV infections is no longer a rare event. Antiviral drugs for the treatment of HSV infections have been developed over the past 40 years. However, most drug-resistant HSV isolates have been discovered in laboratories and clinics, which may contribute to the use of a single target (such as viral DNA polymerase) in all current antiviral drugs. The identification of novel strategies for the development of new antiherpetic molecules with different mechanisms of action that are highly effective and exhibit low toxicity against drug-resistant HSV isolates is challenging. Here, we summarise some of the strategies currently in development:

### New target

**Figure 2:**
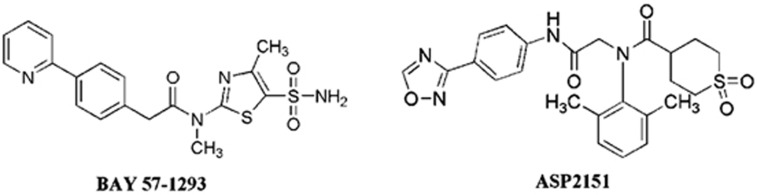
**The chemical structures of two potent HPI active compounds against HSV**. HPI, helicase-primase inhibitor; HSV, herpes simplex virus.

The DNA helicase/primase (H/P) complex is a target for herpes viral infection.^56, 57, 58, 59, 60, 61, 62, 63, 64^ The viral H/P complex is common to all members of the herpes virus family, and it may be a good target for the development of novel anti-HSV agents. The HSV-1 H/P complex includes three components (UL5, UL52, and UL8) that exhibit 5′–3′ helicase, primase, and single-stranded DNA-dependent NTPase activities, respectively. The new inhibitors of the H/P complex have diverse chemical structures, such as thiazole, thiazoleurea, and thiazolyphenyl derivatives.^63^ BAY 57-1293 exhibits almost 200 times greater potency against HSV than ACV *in vitro*^65, 66, 67^ (Figure 2). ASP2151 has been shown to be a safe and effective treatment for genital HSV in Phase III clinical trials^59, 62, 68^ (Figure 2). Some promising compounds have been identified, and several of these compounds have undergone clinical trials. However, several problems still exist. For example, the Phase I clinical trial of ASP2151 was terminated because of adverse events.^69^ Therefore, the development of this new type of drugs will require substantial work in the future.

### New types of molecules

Natural products are an important source of new molecules for use as anti-HSV agents, such as flavonoids, sugar-containing compounds, and peptides.^6, 70, 71, 72^ Researchers have recently found that the notoginsenoside ST-4 inhibits the entry of HSV into cells *in vitro*, with concentrations for 50% of maximal effect (EC_50_s) of 16.47 μmol·L^−1^ and 19.44 μmol·L^−1^ for HSV-1 and HSV-2, respectively.^73, 74^ Cheng and colleagues^75, 76^ have found that putranjivain A and pterocarnin A (from Euphorbia jolkini and pterocaryastenoptera, respectively) inhibit the entry and replication of viruses at concentrations of 2–8 μmol·L^−1^. In the 1990s, Perry *et al.*^77^ first reported that mycalamide A display antiviral activities. Mycalamide A has recently been shown to inhibit HSV-1 at 5 ng per disc.^78^ Traditional Chinese medicine theory (in which one compound may target several proteins, or several compounds may target one protein) has allowed identification of a large number of natural products that inhibit HSV effectively; these discoveries may hopefully solve the present problem of drug resistance. However, the development of natural antiviral drugs faces several challenges, such as the isolation and identification of the active components from complex products, large-scale production, and selective inhibition.

### New antiviral mechanism

**Figure 3:**
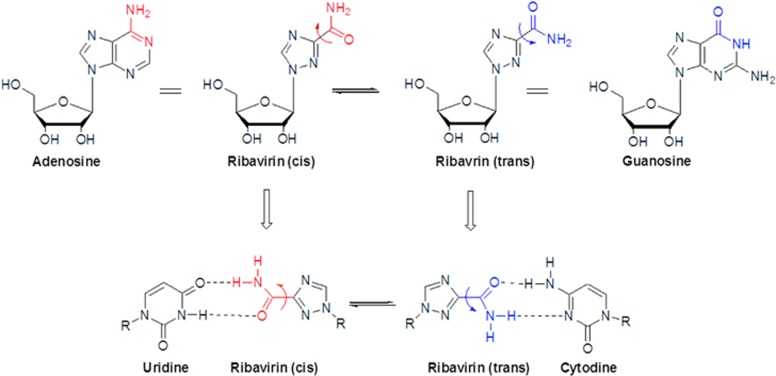
**The lethal mutagenesis mechanism of ribavirin**. The ribavirin *cis* conformer can pair with uridine by mimicking adenosine, and the *trans* conformer can pair with cytidine by mimicking guanosine.

The lethal mutagenesis antiviral mechanism has been proposed as a novel chemotherapeutic strategy for drug resistance.^79, 80, 81, 82, 83, 84, 85, 86, 87, 88, 89, 90, 91^ Viruses survive on the basis of quasispeciestheory,^92, 93^ which states that viruses must maintain high levels of potentially beneficial mutations to adapt to new environments quickly *via* immune responses and antiviral drug therapy. However, the high frequency of mutations in the viral genome also implies a large danger of genetic phenomena. There is an intrinsic limit to the maximum number of mutations in a viral genome before the virus loses its infection activities. The viral genetic information may be lost if the virus quasispecies exceeds the limitation, or it may result in a lethal accumulation of errors (termed lethal mutagenesis). Therefore, lethal mutagenesis may be effective not only in reducing viral infection activity but also in weakening the capacity of the virus for drug resistance. Only one nucleoside analogue, ribavirin, exhibits broad spectrum of antiviral activity against DNA- and RNA-based viruses. Ribavirin is also a classic example that is mutagenic in viral cell cultures. Crotty and colleagues^79, 80, 90, 94, 95^ have demonstrated that ribavirin may be a template for uridine or cytidine with equal efficiency *via* rotation around the C^3^-carbonyl bond to give *s-cis* and *s-trans* conformers, which may have pushed the viral genome mutations beyond the error threshold (Figure 3).

However, the efficiency of ribavirin's incorporation into a viral genome is relatively low. The exploration of new mutagenic molecules to efficiently lead to the mutation of a viral genome is an excellent strategy to develop new antiviral drugs on the basis of lethal mutagenesis. Numerous researchers have focused on advancing the application of nucleoside molecules to induce viral lethal mutations^88, 91^ (Table 2). For example, 5-aza-5,6-dihydro-2′-deoxycytidine (KP-1212) pairs with different natural purines (guanosine and adenosine) by the diverse tautomerization of the nucleobase (amino and imino).^96, 97, 98^ KP-1212 inhibits HIV with an EC_50_ of 10 nmol·L^−1^, which increases the mutation frequency of proviral HIV-1 DNA by 50%–100% and does not result in resistance or genotoxicity to the host.^99^ The prodrug of KP-1212, KP-1461, has been used as a monotherapy for the treatment of HIV-1 infection with significant resistance in Phase IIa clinical trials, which have provided critical insight for the translation to clinical use and new avenues for drug development.^91, 100^

**Figure 4:**
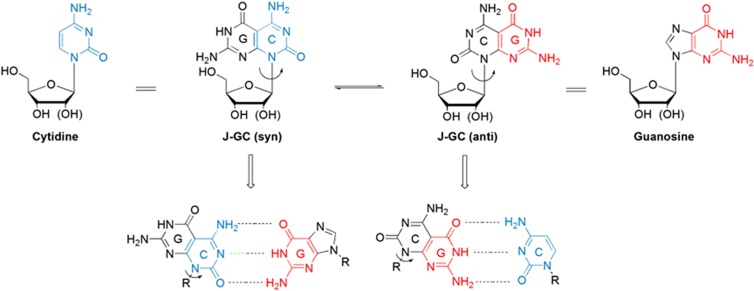
**The potential mutagenic molecule**. Janus nucleoside analogues (for example, J-GC) can pair with guanosine and cytidine by rotating around the glycosyl bond. J-GC, Janus-type pyrimido[4,5-d]pyrimidine guanosine–cytosine.

Two different faces/base-pairing systems, a novel Janus-type pyrimido[4,5-d]pyrimidine guanosine–cytosine (J-GC) ribonucleoside and 2′-deoxyribosenucleoside with a tridentate hydrogen bonding pattern, have been designed and synthesised for lethal mutagenesis.^101, 102^ Cristol first proposed Janus molecules (from the two-faced Roman god Janus) to describe a new symmetrical carbocyclic system. The J-GC mimics natural nucleosides and has the structure of canonical pyrimidine and pure systems in a single molecule (Figure 4). The Watson–Crick base-pairing pattern of J-GC can maintain the tridentate H-bond array. The base moiety of J-GC has one face with a Watson–Crick donor–donor–acceptor H-bond pattern of guanine and the other face with an acceptor–acceptor–donor array of cytosine. The J-GC has two different conformations (*syn* or *anti*), which allow pairing with diverse nucleosides in the viral genome *via* rotation around the glycosyl bond and further induces viral lethal mutations, in a similar manner to ribavirin (Figure 4).

Janus-type pyrimido[4,5-d]pyrimidine adenosine–thymidine (J-AT) nucleosides have been synthesised to expand this tridentate J-GC nucleoside system to a bidentate J-AT nucleoside system and obtain a combination of all four chemical letters of the genetic nucleoside alphabet.^103^ The base moiety of J-AT has one face with a Watson–Crick H-bond acceptor–donor pattern of thymidine and the other face with a donor–acceptor pattern of adenine. J-AT may be able to pair with diverse nucleosides in the viral genome by rotation around the glycosyl bond. Different mono-substituted nucleosides have been synthesised by replacing one N–H on the thymine ring or the adenine ring with corresponding sugar residues attaching to N1, N3, or N8 of a Janus-type adenosine–thymidine system through divergent synthetic routes, such as Vorbruggen or transglycosylation reactions.^104, 105, 106, 107, 108^ The preliminary antiviral activity testing has demonstrated that the J-GC ribonucleoside is active against the hepatitis B virus, which supports the application of Janus-type nucleosides in the field of drug-resistant HSV and the great potential for antiviral drug development. These researchers have also found that the Janus-type nucleosides form different morphogenesis nanostructures (flower-like superstructures, nanobundles, and nanoparticles) by self-assembling in solutions and have demonstrated that the novel self-assembled nucleoside nanoparticle can efficiently act as a drug delivery system in the treatment of oral cancer.^107, 108^ These molecules for the development of this theory for antiviral use are just beginning. However, this subject will likely yield the best advances in strategies against drug resistance.

## Conclusion

ACV and related nucleoside analogues have been gold standard molecules for the treatment of HSV infections during the past decades. However, the emergence of ACV drug-resistant HSV is rising rapidly with the increasing numbers of transplant and cancer patients. Therefore, new antiviral drugs with different antiviral actions, including new antiviral targets, new antiviral mechanisms, and new antiviral molecules, are required. Janus-type nucleosides have two different faces (mimicking the natural purine and pyrimidine systems) in one molecule, and these drugs may pair with diverse natural bases *via* rotation around the glycosyl bond, which further induces viral lethal mutation. Therefore, unique Janus-type nucleoside analogues possess great potential in the exploitation of new lethal mutagenesis drugs as novel strategies for antiviral chemotherapy.

## Figures and Tables

**Table 1:** Three classes of drugs approved for the treatment of HSV infections

Class of drugs	Licensed drugs
Acyclic guanosine analogues	Acyclovir, Ganciclovir, Penciclovir, Valaciclovir, Valganciclovir, Famciclovir
Acyclic nucleotide analogues	Cidofovir, Adefovir dipivoxil
Pyrophosphate analogues	Foscarnet

HSV, herpes simplex virus.

**Table 2:** Selected nucleoside analogue viral mutagens

Chemical name	Mutation
5-aza-5,6-dihydro-2′-deoxycytidine	C/T–U transitions
5-hydroxycytidine	C/T–U transitions
5-aza-2′-deoxycytidine	C/G transversions
2-amino-N6-hydroxyadenosine	A/G transitions
8-oxiguanosine	G/U transversions
